# Association of serum calcium and metabolically healthy obese in US adults: a cross-sectional study

**DOI:** 10.1080/07853890.2024.2403721

**Published:** 2024-09-18

**Authors:** Zhanbin Li, Zhenyu Yao, Qiaoran Liu

**Affiliations:** a Department of Endocrinology, Shandong Provincial Hospital, Shandong University, Key Laboratory of Endocrine Glucose & Lipids Metabolism and Brain Aging, Ministry of Education, Jinan, China; b Shandong Clinical Research Center of Diabetes and Metabolic Diseases, Jinan, China; c Shandong Institute of Endocrine and Metabolic Diseases, Jinan, China; d Department of Breast Surgery, Shandong Provincial Qianfoshan Hospital, Shandong University, Jinan, China

**Keywords:** Metabolically healthy obesity (MHO), metabolically unhealthy non-obese (MUNO), obesity, serum calcium, Nutrition Examination survey (NHANES)

## Abstract

**Objectives:**

The relationship between serum calcium and occurrence of MHO (metabolically healthy obesity) and MUNO (metabolically unhealthy non-obesity) remains unclear, and distinguishing these two phenotypes is difficult within primary healthcare units. This study explores that relationship.

**Methods:**

This survey included 28590 adults from the National Health and Nutrition Examination Survey (NHANES) 2001–2018. Obesity phenotypes were categorized based on BMI and presence or absence of metabolic syndrome components. Weighted multivariate logistic regression analyses were used to assess the association between serum calcium levels and the obesity phenotype. Restricted cubic spline analysis characterized dose-response relationships, and stratified analyses explored these relationships across sociodemographic and lifestyle factors.

**Results:**

The overall prevalence of MHO and MUNO were 2.6% and 46.6%, respectively. After adjusting for covariates, serum calcium exhibited a negative association with MHO [OR (95%): 0.49 (0.36,0.67), *p* < 0.001], while exhibiting a positive association with MUNO [OR (95%): 1.48 (1.26,1.84), *p* < 0.001]. Additionally, we found a non-linear association between serum calcium levels and the incidences of MHO and MUNO. Stratified analyses demonstrated a strong negative correlation between serum calcium levels and MHO occurrence across various subgroups. There was no significant interaction between calcium and stratified variables except sex; the association between calcium and the occurrence of MHO was remarkable in female patients. Meanwhile, the predictive ability of serum calcium level for the occurrence of MUNO among all patients was consistent across various subgroups. There was a significant interaction between calcium level and stratified variables based on age, sex, race, and smoking status; the association was remarkable in older (≥ 40 years old), white, none or less smoking, and female patients.

**Conclusions:**

A significant correlation was identified between serum calcium levels and MHO or MUNO. The findings suggest that serum calcium levels may serve as an indicator for more accurate assessment and diagnosis of MUNO and MHO, especially among individuals with abdominal obesity.

## KEY MESSAGES

1. Serum calcium levels exhibited an inverse relationship with metabolically healthy obesity (MHO) and a positive relationship with metabolically unhealthy non-obese (MUNO).
2. A nonlinear association exists between serum calcium levels and the incidence of both MHO and MUNO.
3. Serum calcium has the potential to enhance evaluation and screening for MUNO or MHO in the general US adult population.

## Introduction

Obesity, a major global public health threat, has surged in recent decades [1], leading to hypertension, type 2 diabetes, and metabolic syndromes [2]. Although obesity is associated with an increased risk of mortality and cardiovascular diseases (CVD) [3], not all obesity phenotypes pose the same risk [4]. While BMI remains a widely employed metric for assessing obesity, its limitations in accurately evaluating obesity-related health risks are undeniable [5–7]. Therefore, accurately identifying metabolic abnormalities within obesity becomes crucial for a comprehensive risk assessment [8–10]. Consequently, individuals can be categorized into four types: metabolically healthy obese (MHO), metabolically unhealthy obese (MUO), metabolically healthy non-obese (MHNO), and metabolically unhealthy non-obese (MUNO), according to their BMI and metabolic states. MHNO is widely recognized as a healthy phenotype, while MUO is considered an unhealthy phenotype. In recent years, studies on two additional phenotypes, MHO and MUNO, have gradually gained momentum. MHO individuals exhibit favorable metabolic characteristics [11–16] and reduced adipose tissue inflammation [16], resulting in a significantly lower risk of cardiovascular complications compared to MUO individuals [11, 17]. In contrast, those with the MUNO phenotype display heightened levels of insulin resistance, blood pressure, oxidative stress [11, 18–20], and atherogenic lipid profiles, which ultimately contribute to unfavorable cardiovascular outcomes [21–23]. Consequently, MHO individuals may receive downgraded health advice and treatment compared with MUO, whereas MUNO should have upgraded health advice and treatment compared with MHNO. Given this, simply assessing risks based on BMI and offering health guidance is indeed sufficient for MHNO and MUO individuals. However, for MHO and MUNO individuals, relying solely on BMI as a metric to evaluate obesity-related risks can potentially lead to misguided health guidance and treatments. Therefore, the early identification of MHO and MUNO individuals holds paramount importance in mitigating the risk of obesity-related complications.

The classification of these phenotypes, especially MHO and MUNO, requires comprehensive clinical evaluation, including the measurement of metabolic markers such as body weight, blood glucose, blood lipids, and blood pressure. Early identification of these phenotypes is critical to prevent, delay, or reduce the progression of metabolic abnormalities. However, these comprehensive tests are often difficult to achieve in primary healthcare units. Considering the inaccuracy of BMI alone in classifying MHO and MUNO phenotypes, and the limitations to carry out comprehensive tests in primary healthcare units, there is an urgent need to develop fast and inexpensive indicators to distinguish between MHO and MUNO.

Research interest is growing in the value of serum ions owing to their cost-effectiveness, rapidity, and convenience of blood testing. Among serum ions, calcium is crucial for numerous physiological functions including muscle contraction, nerve transmission, intracellular signaling, and hormonal secretion [24]. Serum calcium levels are strictly controlled and are not directly correlated with calcium intake, making it a reliable marker of physiological and pathological conditions [25]. Meanwhile, numerous studies have shown that serum calcium levels are associated with BMI [26–28], blood lipid profiles [29], insulin sensitivity [30], diabetes [31], hypertension [32], metabolic syndrome [33, 34], and cardiovascular disease [35, 36]. Given these characteristics, serum calcium level may be a convenient and inexpensive routine clinical indicator for evaluating obesity phenotypes.

However, to the best of our knowledge, the variation in serum calcium levels among obesity phenotypes remains poorly characterized, particularly in the US population. The present study aimed to investigate the prevalence of MHO and MUNO among US adults, exploring the predictive value of serum calcium levels in relation to these obesity phenotypes, and seeking to unravel their intricate relationships in this population.

## Methods

### Study population

The NHANES is a nationally representative, ongoing health survey of non-institutionalized U.S. civilians, conducted biennially by the National Center for Health Statistics (NCHS) since 1999. It combines interviews and medical examinations to gather comprehensive data on demographics, socioeconomic status, diet, physiology, and laboratory tests. All the original data analyzed in this study was obtained from the National Health and Nutrition Examination Survey (NHANES). The NHANES Investigation Protocols [NHANES 1999-2004: Protocol #98-12; NHANES 2005-2010: Protocol #2005-06; NHANES 2011-2018: Protocol #2011-17, #2018-01 (Effective beginning October 26, 2017)] gained approval from the NCHS Research Ethics Review Committee. Moreover, all participants provided written informed consent in accordance with the Declaration of Helsinki. This study used these previously collected deidentified data, and gained approval from the Ethics Committee of the Qianfoshan Hospital Affiliated to Shandong University (2024S882).

**Figure 1.**
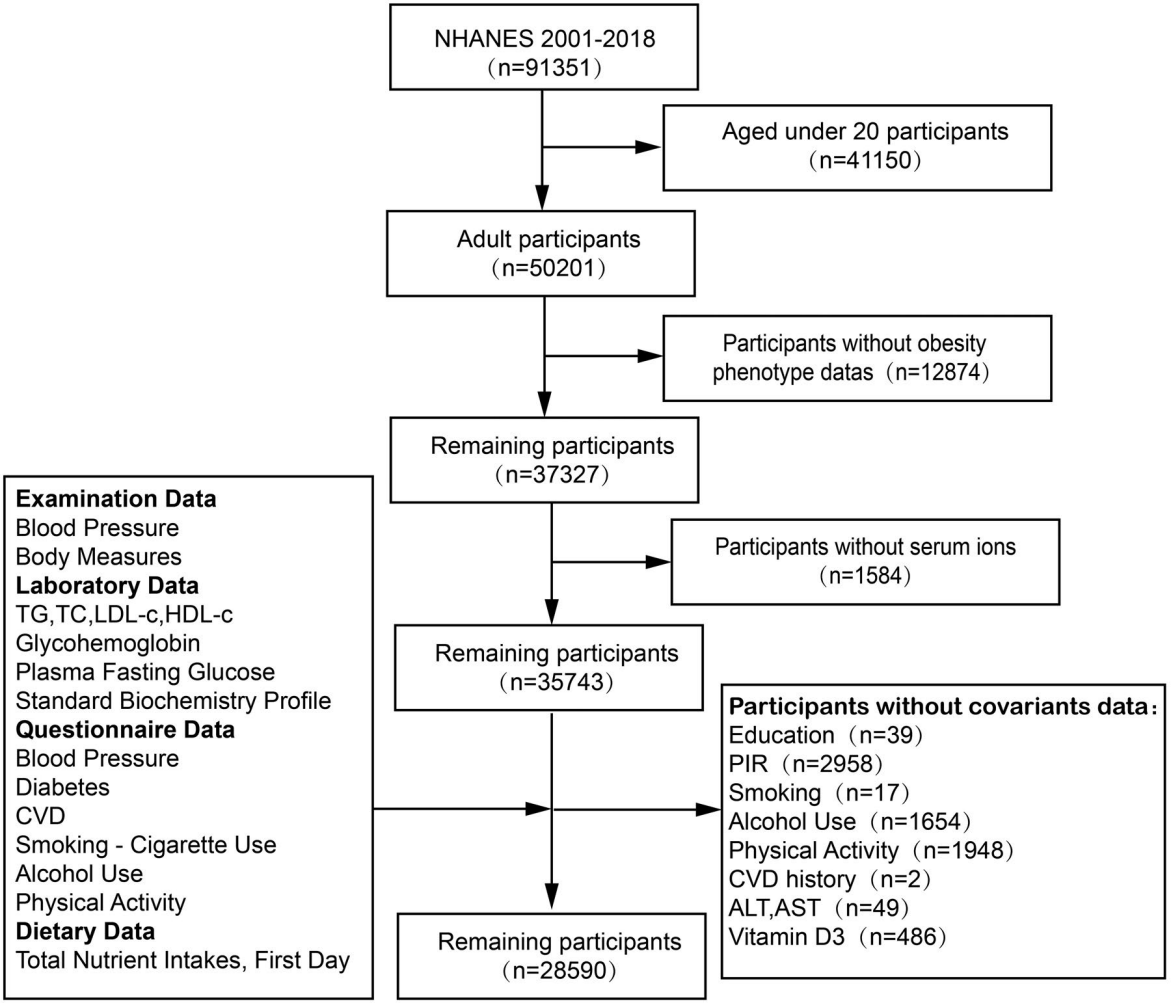
The selection flowchart of the participants.

Utilizing data from 9 consecutive cycles of the NHANES from 2001 to 2018, we constructed a cohort for this study. Participants were excluded for the following reasons: age < 20 years, missing BMI or key metabolic parameter values, and absence of serum calcium, vitamin D3 and dietary calcium intake data. After rigorous exclusion criteria were applied, 28590 patients from the NHANES were included in the final analysis. Figure 1 outlines the detailed inclusion and exclusion process.

### Obesity phenotype criteria

The diagnostic criteria for obesity phenotypes were mainly based on previous studies [37]. Obesity were defined by a BMI ≥ 30.0, while metabolic abnormality was assessed based on the presence of specific conditions: (1) SBP ≥ 130 mmHg, DBP ≥ 85 mmHg, or antihypertensive medication use; (2) FPG ≥100 mg/dL or antidiabetic medication use; (3) high-density lipoprotein cholesterol (HDL-C) <40 mg/dL for men and <50 mg/dL for women; or (4) triglycerides ≥150 mg/dL. Obese participants without metabolic abnormalities were classified as having MHO, whereas those with any abnormalities were designated as having MUO. Adults without obesity or metabolic syndrome components were categorized as MHNO. Conversely, non-obese adults with any metabolic syndrome criteria were labeled MUNO [38–40]

### Serum ions assessment

The standard biochemistry profile in the NHANES database was used to obtain serum ion parameters, including calcium, phosphorus, iron, sodium, potassium, and chloride. Serum specimens were collected from the participants by a phlebotomist at the mobile examination center at the time of recruitment and stored at −20 °C until analysis. The serum total calcium concentrations were assayed using a colorimetric technique, leveraging Roche Hitachi/Beckman Coulter analyzers. According to the NHANES protocol, the normal range for serum levels in adults aged 18–60 is 8.4–10.2 mg/dL. Our analysis identified 91 cases with low blood calcium (<8.4 mg/dL), a minority subset. Our aim is to investigate the calcium-MHO relationship for early identification. To avoid bias, we included this subset in our study, encompassing both normal and abnormal calcium levels. Given that total calcium concentrations fluctuate in tandem with albumin levels, we also used corrected calcium to estimate the free calcium concentration, which was calculated using the following formula: corrected calcium (mg/dL) = serum calcium + [(4.0 − serum albumin) × 0.8] [41]. The Roche method was used for iron and sodium measurement. Phosphorus was measured using ammonium molybdate as the color-forming reagent, while potassium, chloride, and sodium concentrations were measured by electrolyte activity in solution. Details of the laboratory methodology, quality assurance, and data processing can be found on the NHANES website: https://wwwn.cdc.gov/Nchs/Nhanes/2013-2014/BIOPRO_H.htm.

### Covariates

Confounding factors that were potentially associated with obesity were included in this analysis. Information on age, sex, race or ethnicity, education level, and family income was collected from demographic data. Race was categorized as Mexican American, non-Hispanic White, non-Hispanic Black, or other race, whereas education level was categorized as less than high school, high school or equivalent, or college or above. Family economic status was determined using the income to poverty ratio (PIR), with three categories: <1.30, 1.31 to 3.50, and ≥3.50 [42].

Smoking status, alcohol consumption, and physical activity were assessed using standardized questionnaires. Participants were categorized into nonsmokers, former smokers, and current smokers, based on their smoking history and habits. Alcohol consumption was determined using a 24-hour dietary recall, classifying individuals as nondrinkers, moderate drinkers (0.1–27.9 g/day for men, 0.1–13.9 g/day for women), or heavy drinkers (≥28 g/day for men, ≥14 g/day for women). Physical activity was divided into inactive, active (meeting recommended levels), and insufficiently active categories, based on previous literature [43].

The diagnosis of CVD was established through a standardized questionnaire, wherein participants were asked to self-report any physician-diagnosed condition, including CHF, CHD, angina pectoris, MI, or stroke. Affirmative responses to any of these conditions indicate a diagnosis of CVD.

Serum 25(OH)D concentrations were measured by DiaSorin radioimmunoassay kit (Stillwater, MN) in the NHANES III and NHANES 2001–2006, and by a standardized liquid chromatography-tandem mass spectrometry (LC MS/MS) method in NHANES 2007–2014. Serum 25(OH)D data from NHANES III and NHANES 2001–2006 were converted by a regression method to be equivalent 25(OH)D measurements from the LC-MS/MS method (https://wwwn.cdc.gov/nchs/nhanes/vitamind/analyticalnote.aspx). Dietary intake data and data on the calcium derived from individual foods were obtained from the 24 h dietary recall survey data ‘Dietary Interview—Individual Foods’. For survey cycles from 2003–2004 to 2017–2018, when two 24-h dietary recalls were per formed, only the first day’s 24-h dietary recall data was included in this study to keep data consistent across all survey cycles [44].

Clinical indicators such as serum ALB, serum ALT, serum AST, serum BUN, serum creatinine (Scr), fasting blood glucose (FBG), HbA1c, triglycerides (TG), total cholesterol (TC), low-density lipoprotein cholesterol (LDL-C), and high-density lipoprotein cholesterol (HDL-C) were measured in the NHANES laboratory, following the relevant standardized protocols. eGFR was calculated using the Chronic Kidney Disease Epidemiology Collaboration Equation [45].

### Statistical analysis

Statistical analysis was performed using R software (version 4.3.1; https://www.r-project.org). Given the complicated sampling design, NHANES weights and strata variables were considered when calculating statistics [46]. Data were categorized into continuous (mean ± SD) and categorical (percentage) variables. Statistical analysis of continuous variables was performed using the Student’s t-test or Mann–Whitney U test, depending on the data distribution. Categorical variables were compared using the chi-squared test. Stratified obesity phenotypes and baseline characteristics were compared using one-way ANOVA.

To assess the independent predictive value of serum calcium level, we constructed three multivariate weighted logistic regression models to adjust for potential confounders. Model 1 was unadjusted; Model 2 was adjusted for age, race, and sex; and Model 3 was adjusted for age, sex, race, education level, family income level, ALT, AST, BUN, Scr, eGFR, 25(OH)D, Dietary calcium intake, smoking status, alcohol intake, physical activity, and CVD history.

We utilized the restricted cubic spline (RCS) model to demonstrate the dose-response relationship between serum calcium levels and MHO or MUNO levels. Stratified analyses were performed in the strata of age (< 30, 30–40, 40–50 or ≥50 years old), sex (male or female), race or ethnicity (White, Black, Mexican, or Other), education level (less than high school, high school or equivalent, or college or above), PIR (<1.30, 1.31 to 3.50, and ≥3.50), smoking status (current smoker, former smoker or nonsmoker), alcohol consumption (heavy drinking, moderate drinking or nondrinking), physical activity(active, insufficiently or active) and CVD history. *P* significance was set at *p* < 0.05. No missing data for diabetes and hypertension covariates and outcomes were recorded in our dataset.

## Results

### Baseline characteristics of the participants

Table 1 shows the baseline demographic, lifestyle, and medical characteristics of the study participants stratified into obesity phenotype groups. The participants’ mean age was 48.82 ± 16.65 years old, and 50.8% of them were women and 49.2% were men. In terms of race, 8% participants self-identified as Mexican American, 10.2% as non-Hispanic Black, 70.4% as non-Hispanic White, and 11.4% as other races or ethnicities. These results are consistent with previous epidemiological research findings [37]. The overall prevalence of MHO and MUNO were 2.6% and 46.6%, respectively. The average serum calcium was 9.41 ± 0.37 mg/ml.

**Table 1.** Baseline characteristics of participants.

	Overall	Obesity phenotype^a^				*P*
		MHO	MUO	MHNO	MUNO	
N (cases)	120287127.4	3104816.0 (2.6)	46127003.4 (38.3)	14957604.7 (12.4)	56097703.4 (46.6)	
Gender [female (%)]	61089880.5 (50.8)	1923534.6 (62.0)	24184311.2 (52.4)	9009075.0 (60.2)	25972959.7 (46.3)	<0.001
Age (years)	48.82 ± 16.65	38.74 ± 13.3	49.46 ± 15.47	38.49 ± 13.82	51.6 ± 17.17	<0.001
Race (%)						<0.001
Mexican American	9599019.7 (8.0)	305951.0 (9.9)	4175792.3 (9.1)	1007760.0 (6.7)	4109516.5 (7.3)	
Other race	13747946.0 (11.4)	325009.8 (10.5)	4390412.5 (9.5)	1694909.5 (11.3)	7337614.3 (13.1)	
Non–Hispanic White	84663202.9 (70.4)	1964008.0 (63.3)	31770724.9 (68.9)	10983415.7 (73.4)	39945054.3 (71.2)	
Non-Hispanic Black	12276958.8 (10.2)	509847.2 (16.4)	5790073.8 (12.6)	1271519.5 (8.5)	4705518.4 (8.4)	
Education (%)						<0.001
Less than high school	19651506.8 (16.3)	421337.6 (13.6)	7715751.6 (16.7)	1523775.6 (10.2)	9990642.0 (17.8)	
High school	29538951.4 (24.6)	645432.8 (20.8)	12319705.4 (26.7)	2737524.6 (18.3)	13836288.6 (24.7)	
College or above	71096669.2 (59.1)	2038045.6 (65.6)	26091546.4 (56.6)	10696304.4 (71.5)	32270772.8 (57.5)	
PIR (%)						<0.001
<1.30	24859965.9 (20.7)	660600.4 (21.3)	10032122.7 (21.7)	2594105.4 (17.3)	11573137.3 (20.6)	
1.31–3.50	44264711.8 (36.8)	1151567.3 (37.1)	17632280.2 (38.2)	4926189.6 (32.9)	20554674.7 (36.6)	
≥3.50	51162449.8 (42.5)	1292648.3 (41.6)	18462600.5 (40.0)	7437309.7 (49.7)	23969891.4 (42.7)	
Smoking status (%)						<0.001
Current smokers	25570025.3 (21.3)	449010.8 (14.5)	8559373.6 (18.6)	3046044.9 (20.4)	13515595.9 (24.1)	
Former smokers	31738557.0 (26.4)	732543.4 (23.6)	13268447.0 (28.8)	2872109.6 (19.2)	14865457.0 (26.5)	
Non-smokers	62978545.1 (52.4)	1923261.8 (61.9)	24299182.8 (52.7)	9039450.1 (60.4)	27716650.5 (49.4)	
Alcohol consumption (%)						<0.001
Heavy drinking	21779834.1 (18.1)	554256.0 (17.9)	6378384.4 (13.8)	3592434.7 (24.0)	11254759.1 (20.1)	
Moderate drinking	9843736.6 (8.2)	227097.5 (7.3)	2981348.7 (6.5)	1388028.3 (9.3)	5247262.0 (9.4)	
Non-drinkers	88663556.7 (73.7)	2323462.5 (74.8)	36767270.3 (79.7)	9977141.7 (66.7)	39595682.3 (70.6)	
Physical activity (%)						<0.001
Active	34616802.9 (28.8)	1030944.5 (33.2)	10420887.8 (22.6)	6392358.0 (42.7)	16772612.5 (29.9)	
Insufficiently	30548650.9 (25.4)	878832.7 (28.3)	11269685.1 (24.4)	4128588.7 (27.6)	14271544.3 (25.4)	
Inactive	55121673.7 (45.8)	1195038.9 (38.5)	24436430.4 (53.0)	4436657.9 (29.7)	25053546.5 (44.7)	
Diabetes (%)	42200317.3 (53.3)	0.0 (0.0)	20801419.7 (75.8)	0.0 (0.0)	21398897.6 (63.5)	<0.001
Hypertension (%)	63463253.6 (53.3)	0.0 (0.0)	30403480.0 (66.8)	0.0 (0.0)	33059773.6 (59.5)	<0.001
CVD (%)	11694853.1 (9.7)	72629.1 (2.3)	5558336.6 (12.1)	325662.1 (2.2)	5738225.3 (10.2)	<0.001
Calcium (mg/mL)	9.41 ± 0.37	9.27 ± 0.33	9.38 ± 0.37	9.39 ± 0.32	9.44 ± 0.37	<0.001
Corrected-calcium (mg/mL)	9.22 ± 0.34	9.17 ± 0.3	9.26 ± 0.34	9.13 ± 0.28	9.21 ± 0.35	<0.001
Phosphorus (mmol/L)	1.2 ± 0.18	1.17 ± 0.16	1.19 ± 0.18	1.21 ± 0.17	1.2 ± 0.18	<0.001
Iron (μmol/L)	15.57 ± 6.45	15.14 ± 6.22	14.24 ± 5.84	17.3 ± 7.1	16.24 ± 6.55	<0.001
Sodium (mmol/L)	139.21 ± 2.36	139.06 ± 1.9	139.15 ± 2.4	139.21 ± 2.04	139.27 ± 2.43	0.046
Potassium (mmol/L)	4.01 ± 0.34	4.02 ± 0.27	4.02 ± 0.34	4.01 ± 0.31	4.01 ± 0.36	0.793
Chloride (mmol/L)	103.33 ± 3	104.4 ± 2.55	103.34 ± 3.09	103.84 ± 2.48	103.13 ± 3.03	<0.001
Albumin (g/L)	4.24 ± 0.33	4.12 ± 0.34	4.15 ± 0.33	4.33 ± 0.31	4.29 ± 0.33	<0.001
ALT (U/L)	25.97 ± 20.75	24.85 ± 15.69	29.07 ± 23.68	21.68 ± 24.72	24.64 ± 16.46	<0.001
AST (U/L)	25.39 ± 16.93	23.52 ± 14.55	25.94 ± 17.11	23.97 ± 22.29	25.43 ± 15.12	<0.001
BUN (mmol/L)	4.88 ± 1.98	4.36 ± 1.46	4.97 ± 2.12	4.36 ± 1.45	4.97 ± 1.99	<0.001
Scr (μmol/L)	79.33 ± 33.63	72.61 ± 16.05	79.58 ± 35.68	73.79 ± 16.68	80.97 ± 35.74	<0.001
eGFR (mL/min/1.73m2)	98.09 ± 27.57	107.01 ± 25.14	97.59 ± 28.41	105.24 ± 24.35	96.09 ± 27.41	<0.001
Vitamin D3 (nmol/L)	64.97 ± 26.9	60.88 ± 25.57	59.37 ± 25.58	70.89 ± 26.75	68.21 ± 27.21	<0.001
Dietary calcium intake (mg)	75.32 ± 147.66	87.48 ± 157.03	77.95 ± 147.23	84.51 ± 169.62	70.03 ± 140.84	<0.001
PTH (pg/mL)^b^	43.55 ± 23.48	40.06 ± 14.01	47.32 ± 24.03	36.55 ± 16.36	43 ± 24.7	<0.001
BMI (kg/m^2^)	29.64 ± 6.91	34.29 ± 3.91	36.19 ± 5.85	23.77 ± 3.1	25.57 ± 2.93	<0.001
WC (cm)^b^	100.94 ± 16.36	108.19 ± 10.52	115.6 ± 12.87	85.03 ± 9.47	92.87 ± 9.8	<0.001
TC (mmol/L)	196.08 ± 42.69	191.57 ± 34.47	196.21 ± 42.73	187.22 ± 35.17	198.58 ± 44.54	<0.001
TG (mmol/L)^b^	131.12 ± 118.02	87.52 ± 30.4	161.37 ± 140.8	77.92 ± 29.5	138.23 ± 121.55	<0.001
LDL-C (mmol/L)^b^	115.32 ± 35.32	117 ± 31.52	116.42 ± 35.57	107.65 ± 31.53	118.06 ± 36.7	<0.001
HDL-C (mg/dL)	51.58 ± 16.34	57.06 ± 11.21	45.88 ± 12.99	63.97 ± 14.75	52.65 ± 17.27	<0.001
FPG (mmol/L)^b^	105.7 ± 30.49	92.27 ± 5.43	116 ± 37.86	90.43 ± 6.12	106.59 ± 29.29	<0.001
HbA1c (%)	5.66 ± 0.97	5.28 ± 0.31	5.92 ± 1.14	5.2 ± 0.3	5.59 ± 0.89	<0.001

^a^ Values are mean (standard deviation) for continuous variables and percentages for categorical variables.

^b^ Numbers may not sum up to the total number of participants due to missing data.

The trend analysis of the MHO proportion was limited to obese adults. The mean age was 48.78 ± 15.56 years old and 53% were women. The prevalence of MHO is 6.3%. Compared with participants with MUO, participants with MHO were more likely to be younger, Mexican or Black, female, and have fewer comorbidities. Participants with the MHO phenotype had significantly lower calcium levels than those with the MUO phenotype did. Oppositely, among the non-obese subjects (59%), the mean age was 48.84 ± 17.36 years old and 49.2% were women. The prevalence of MUNO was 78.9%. Participants with MUNO were more likely to be older, male, and have more comorbidities than those in the MHNO group. Participants with the MUNO phenotype had significantly higher serum calcium levels.

### The association of serum ions with MHO and MUNO

The results of the logistic regression analysis are presented in Table 2. The results indicate that after adjusting for covariates, we found serum calcium level exhibited a negative association with MHO in its continuous variable [OR (95%): 0.49 (0.36,0.67), *p* < 0.001]. When analyzed as categorical variable, the highest quartile of serum calcium [OR (95%): 0.51 (0.36,0.73), *p* < 0.001] was related to lower incidence of MHO. Oppositely, after adjusting for covariates, serum calcium exhibited a positive association with MUNO in its continuous variable [OR (95%): 1.48 (1.27,1.72), *p* < 0.001], and the highest quartile of serum calcium [OR (95%): 1.52 (1.26,1.84), *p* < 0.001] was associated with higher incidence of MUNO in categorical variables. Furthermore, after adjusting serum calcium levels for albumin, the significant trends persisted. Upon screening the NHANES 2003–2006 dataset with ­parathyroid hormone data and making adjustments, statistical significance of the differences was retained (Table S1). These results suggest that serum calcium level is a good candidate for screening different obesity phenotypes.

**Table 2.** Associations of serum calcium concentrations with MHO and MUNO in US adults.

Independent variables	Mode 1			Mode 2			Mode 3		
	OR	95%CI	*P*	OR	95%CI	*P*	OR	95%CI	*P*
Incidence of MHO									
Calcium	0.44	0.34,0.58	<0.001	0.49	0.37,0.65	<0.001	0.49	0.36,0.67	<0.001
Q1	Reference								
Q2	0.9	0.71,1.13	0.352	0.95	0.74,1.22	0.679	0.96	0.74,1.23	0.733
Q3	0.51	0.37,0.69	<0.001	0.54	0.39,0.75	<0.001	0.55	0.4,0.77	0.001
Q4	0.45	0.31,0.63	<0.001	0.5	0.35,0.71	<0.001	0.51	0.36,0.73	<0.001
*P* for trend			<0.001			<0.001			<0.001
Corrected-calcium	0.47	0.35,0.63	<0.001	0.45	0.33,0.63	<0.001	0.49	0.35,0.69	<0.001
Q1	Reference								
Q2	0.76	0.59,0.98	0.036	0.74	0.56,0.96	0.026	0.74	0.56,0.98	0.034
Q3	0.71	0.53,0.94	0.019	0.67	0.5,0.89	0.006	0.69	0.51,0.93	0.016
Q4	0.49	0.35,0.67	<0.001	0.49	0.35,0.69	0	0.52	0.37,0.73	<0.001
*P* for trend			<0.001			<0.001			<0.001
Incidence of MUNO									
Calcium	1.56	1.37,1.78	<0.001	1.48	1.28,1.71	<0.001	1.48	1.27,1.72	<0.001
Q1	Reference								
Q2	0.9	0.78,1.06	0.202	0.91	0.78,1.07	0.27	0.92	0.78,1.07	0.277
Q3	1.15	1,1.33	0.057	1.14	0.97,1.33	0.104	1.13	0.97,1.32	0.106
Q4	1.59	1.35,1.87	<0.001	1.54	1.28,1.84	<0.001	1.52	1.26,1.84	<0.001
*P* for trend			<0.001			<0.001			<0.001
Corrected-calcium	2.21	1.92,2.55	<0.001	1.8	1.53,2.12	<0.001	1.63	1.38,1.94	<0.001
Q1	Reference								
Q2	1.12	0.98,1.27	0.084	1.07	0.93,1.23	0.372	1.04	0.9,1.2	0.613
Q3	1.43	1.25,1.64	<0.001	1.28	1.1,1.5	0.002	1.23	1.05,1.44	0.012
Q4	2.07	1.78,2.4	<0.001	1.76	1.49,2.07	<0.001	1.59	1.33,1.89	<0.001
*P* for trend			<0.001			<0.001			<0.001

Note: Multivariate weighted logistic regression models with three models to control for confounding factors.

Model 1 was unadjusted.

Model 2 was adjusted for age, race, and gender.

Model 3 was adjusted for age, sex, race, education level, family income level, serum ALT, AST, BUN, Scr, eGFR, Vitamin D3, Dietary calcium intake, smoking status, alcohol intake, physical activity, and CVD.

Interestingly, the results demonstrated the risk of MHO was negatively associated with phosphorus [OR (95%): 0.42 (0.25,0.71), *p* = 0.001] and positive associated with iron [OR (95%): 1.05 (1.03,1.06), *p* < 0.001] and chloride [OR (95%): 1.1 (1.06,1.14), *p* < 0.001]. Conversely, a negative association between iron [OR (95%): 0.98 (0.97,0.99), *p* < 0.001], chloride [OR (95%): 0.94 (0.92,0.96), *p* < 0.001], and incidence of MUNO (Table S2).

### The dose-response association of serum calcium with MHO or MUNO

**Figure 2.**
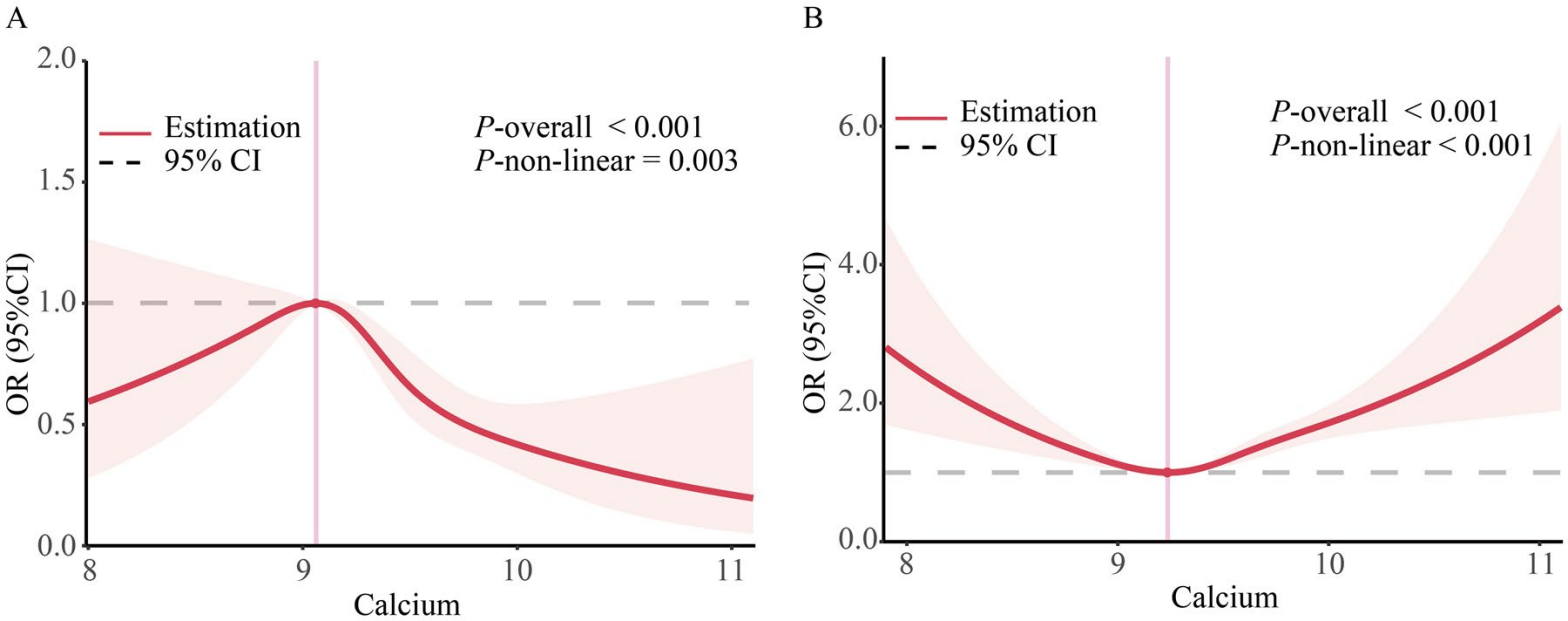
Dose-response associations of serum calcium with incidence of MHO and MUNO in US adults. Association between serum calcium with incidence of MHO (A) and MUNO (B) in US adults. The associations were examined by generalized additive models with restricted cubic splines. ORs adjusted for Model 3 (age, sex, race, education level, family income level, serum ALT, AST, BUN, Scr, eGFR, Vitamin D3, Dietary calcium intake, smoking status, alcohol intake, physical activity, and CVD). Solid lines represent estimates of ORs and dashed lines represent 95% CIs.

Using generalized additive models and restricted cubic spline analysis, we further explored the correlation between serum calcium and the risk of MHO or MUNO. After adjusting for multiple potential confounders, we discovered that the adjusted smoothed plots displayed an inverted U-shaped and U-shaped association between serum calcium and the incidence of MHO (*P*-nonlinearity = 0.003) (Figure 2A) and MUNO (*P*-nonlinearity < 0.001) (Figure 2B). We discovered that the inflection points for MHO and MUNO were 9.059 and 9.235 mg/mL, respectively. When serum calcium concentrations were exceeded 9.059 and 9.235 mg/mL, the adjusted OR of calcium for MHO [OR (95%): 0.32 (0.19,0.55), *p* < 0.001] and MUNO [OR (95%): 2.57 (1.84,3.6), *p* < 0.001] increase significantly. When serum calcium concentrations were < 9.059 mg/mL and 9.235 mg/mL, there was no association with MHO or MUNO (Table 3).

**Table 3.** Threshold effect analysis of serum ions on incidence of MHO and MUNO in US patients.

MHO	Adjusted OR	95% CI	*P* value
Calcium	0.49	0.36,0.67	<0.001
<9.059	1.31	0.3,5.78	0.718
≥9.059	0.32	0.19,0.55	<0.001
MUNO	Adjusted OR	95% CI	*P* value
Calcium	0.48	1.27,1.72	<0.001
<9.235	0.68	0.4,1.17	0.164
≥9.235	2.57	1.84,3.6	<0.001

Note: Logistic regression models were used to estimate OR and 95% CI adjusted for age, sex, race, education level, family income level, serum ALT, AST, BUN, Scr, eGFR, Vitamin D3, Dietary calcium intake, smoking status, alcohol intake, physical activity, and CVD.

### Stratified analyses

**Figure 3.**
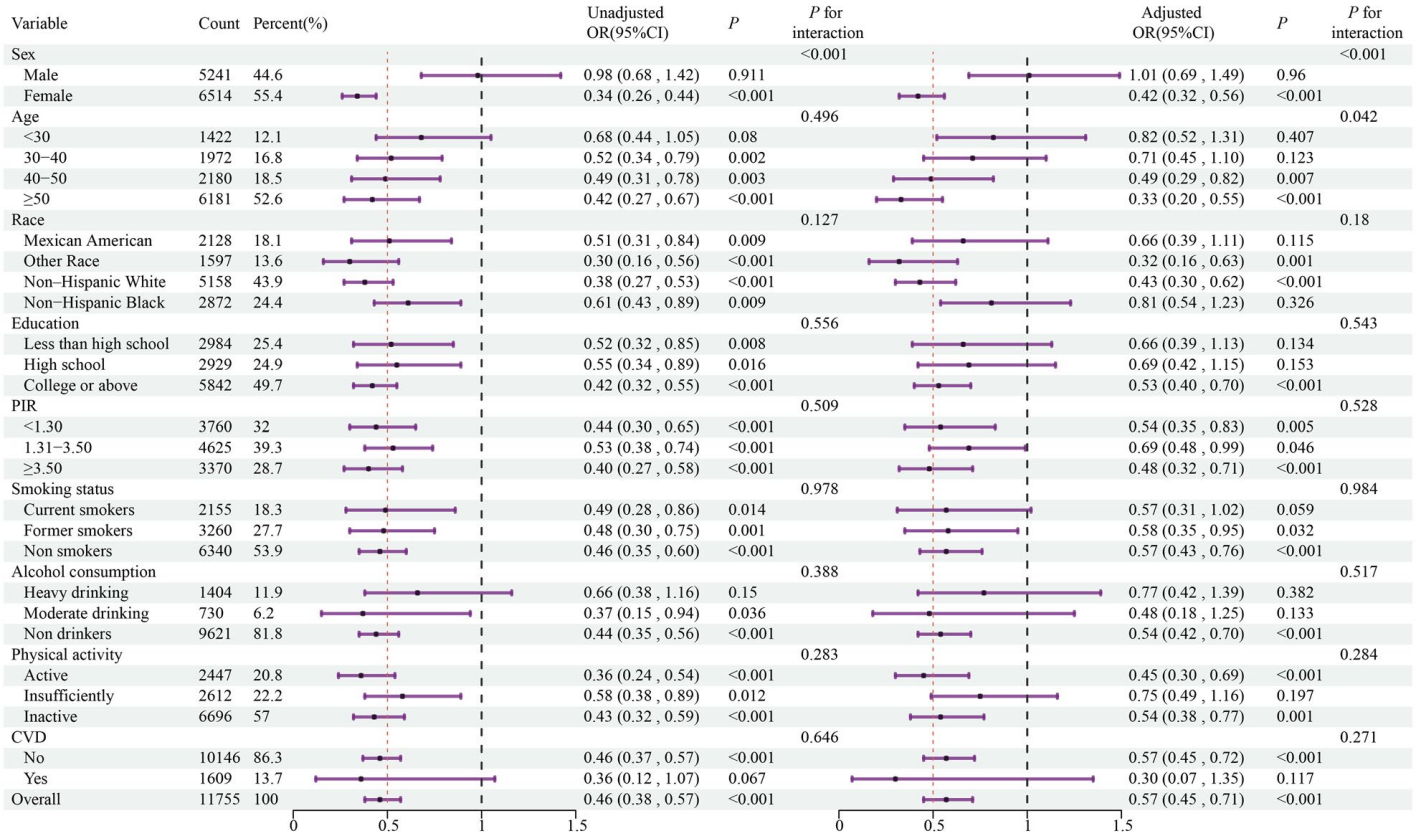
Stratified analyses for the association between serum calcium level as a continuous variable and incidence of MHO. Odds ratio adjusted for variables in the Model 3 (age, sex, race, education level, family income level, serum ALT, AST, BUN, Scr, eGFR, Vitamin D3, Dietary calcium intake, smoking status, alcohol intake, physical activity, and CVD) except the corresponding stratification variable.

**Figure 4.**
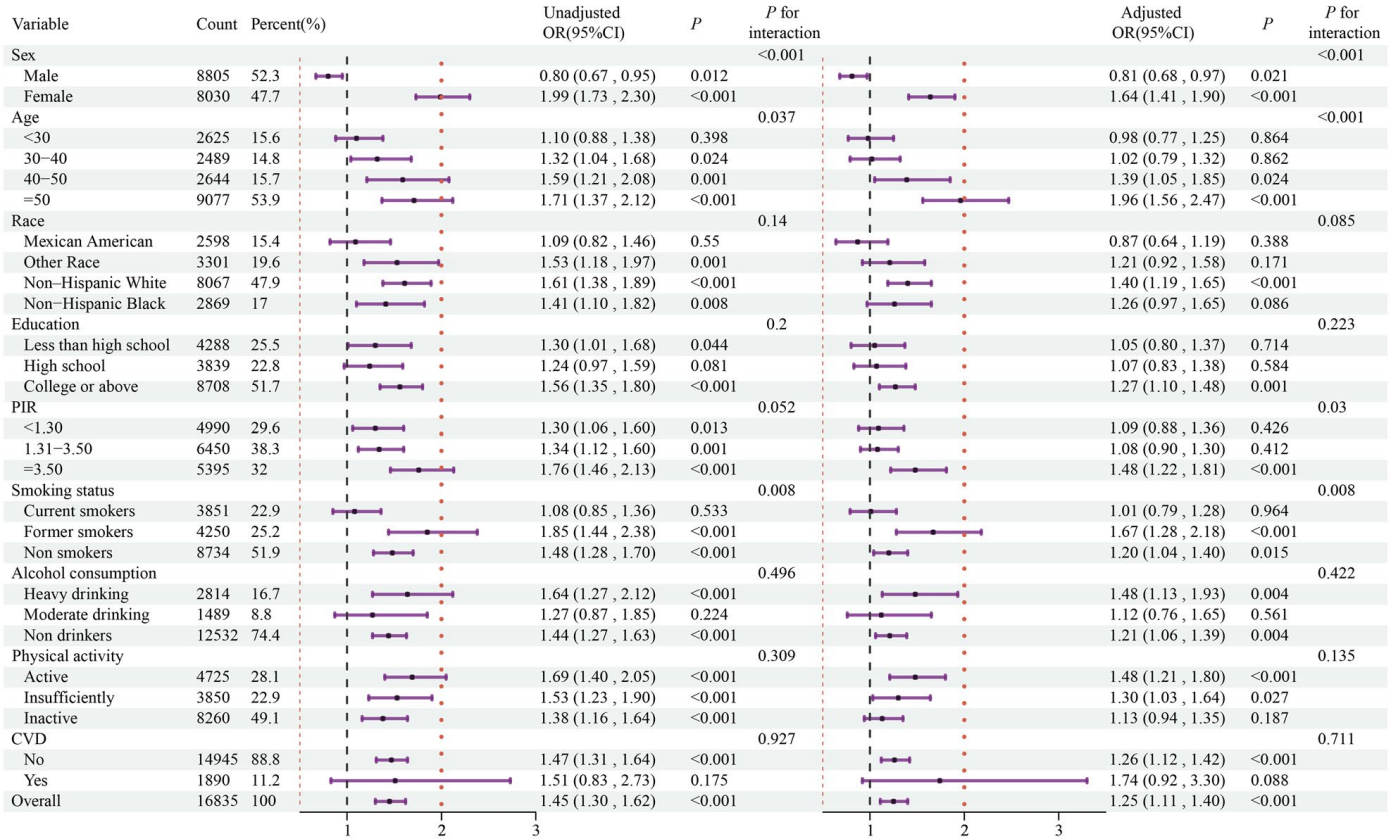
Stratified analyses for the association between serum calcium level as a continuous variable and incidence of MUNO. Odds ratio adjusted for variables in the Model 3 (age, sex, race, education level, family income level, serum ALT, AST, BUN, Scr, eGFR, Vitamin D3, Dietary calcium intake, smoking status, alcohol intake, physical activity, and CVD) except the corresponding stratification variable.

The results of the stratified analyses based on age, sex, race, education level, family income level, smoking status, alcohol intake, physical activity and CVD history are shown in Figures 3 and 4. The ability of serum calcium level to predict the occurrence of MHO among obese patients was consistent across various subgroups. There was a significant interaction between the baseline calcium level and stratified variables based on sex, and the association between calcium and the occurrence of MHO was remarkable in female patients, but not in male patients. Meanwhile, our findings showed a stronger inverse association between calcium and the occurrence of MHO in older (≥ 40 years old) patients.

In contrast, the predictive ability of serum calcium level for the occurrence of MUNO among non-obese patients were consistent across various subgroups, as depicted in Figure 4. There was a significant interaction between serum calcium level and stratified variables based on sex, age, race, and smoking status. The association between calcium and the occurrence of MUNO was remarkable in older (≥ 40 years old), white, none or less smoking, and in female patients. There was no significant interaction between the baseline calcium levels and other stratified variables.

## Discussion

To our knowledge, this is the first study to investigate the relationship between the serum calcium with obesity phenotypes among the general population, the results demonstrated that the overall prevalence of MHO and MUNO was 2.6% and 46.6% in general population. Among the subjects with obesity, the prevalence of MHO was 6.3%, while the prevalence of MUNO was 78.9% in adults without obesity. As we explored the intricacies leading to obesity phenotypes, the current study revealed that serum calcium appears to be inversely associated with the risk of MHO among obese patients, while positively correlating with the incidence of MUNO among non-obese patients from the NHANES 2001–2018 dataset. Additionally, we revealed that one inflection point for MHO and MUNO was 9.059 and 9.235 mg/ml, respectively. Rigorous regression and stratified analyses were conducted to guarantee the credibility and robustness of our findings, ensuring high reliability and accuracy. Our study shows that serum calcium level is a strong indicator of MHO and MUNO in obese and non-obese adults in the US.

Our study showed a lower incidence of MHO and a higher incidence of MUNO compared to two prior cohort studies on US adults. One comprising 4291 participants from Framingham Offspring Cohort reported that 4% and 31% of participants exhibited the MHO and MUNO sub-phenotypes [47]. The other study including 20430 US adult participants found that MHO prevalence among US adults was 3.2–6.6% across years and 10.6–15.0% among the population with obesity [37]. Multiple studies across various populations have demonstrated significant variation in the prevalence of MHO. In cohorts from multiple European countries, the prevalence of MHO ranges from 7% to 28% among women and from 2% to 19% among men [48]. One cohort study comprising 21121 Russian population reported MHO phenotype was 41.5% in obese people, whereas MUNO phenotype was 34.4% in non-obese subjects [38]. Another study enrolled 514866 participant from Korean National Health Insurance Service-National Sample Cohort found 10.0% and 32.2% of the subjects were categorized as MHO and MUNO [49]. The Studies from Chinese adult population reported that the prevalence of MHO and MUNO have been shown to range between 4.2–13.6% and 22.7–49% [50, 51]. The underlying reasons for the inconsistency are complex because of the differences in the study population, sample size, inclusion criteria, and different MHO definitions. This also suggests that most studies have overestimated the prevalence of MHO and underestimated the prevalence of MUNO. In the current study, we used strict criteria[37], controlled for various potential confounders. Our findings lend strong epidemiological support to the association between serum calcium levels and obesity phenotypes.

Despite the absence of direct research exploring the link between serum calcium levels and obesity phenotypes, extensive studies have established a significant association between serum calcium levels and obesity as well as metabolic syndrome. Researchers have documented a significant independent association between serum calcium levels and obesity [27, 28]. Cross-sectional and longitudinal studies have consistently reported a decreased risk of metabolic syndrome with higher serum calcium levels [33, 52–56]. Additionally, serum calcium levels are tightly linked to each component of the metabolic syndrome. Epidemiological studies further suggest that higher calcium levels are associated with insulin resistance [54, 57, 58], type 2 diabetes [59], hypertension [32], as well as well as hypercholesterolemia[60]. These findings offer insights into the link between serum calcium levels and obesity phenotype.

Serum calcium levels are influenced by myriad factors, and the underlying mechanisms are complex and challenging to fully elucidate. One postulated mechanism suggests that calcium may play a role in lipid accumulation and lipolysis in preadipocytes *via* the p38 MAPK pathway [61]. Although this provides a potential explanation, the exact mechanism remains unclear and requires further investigation.

Interestingly, we discovered associations between serum iron and chlorine levels and obesity phenotypes. Consistent with our observations, prior research has indicated that iron is linked to obesity [62–65], glucose, and lipid homoeostasis [66]. Additionally, some studies have demonstrated a positive association between hypochloremia and obesity [67–69] as well as hypertension [70]. However, the precise mechanisms underlying this relationship remain unclear. A comprehensive understanding of the mechanisms that connect ion metabolism with metabolic risk factors could pave the way for future therapeutic intervention. Therefore, further exploration in this area is necessary.

To fully appreciate the research findings, acknowledging the limitations of this cross-sectional study is crucial. First, causality could not be definitively established, necessitating further cohort studies to confirm these results. Second, although valuable, cross-sectional studies are susceptible to confounding variables that could potentially bias the results, thereby affecting the interpretation of findings. Although attempts have been made to account for these factors, unknown variables or biases may still exist, leading to inaccurate results. Therefore, a cautious approach is warranted when interpreting the findings, which requires further validation under different conditions. Lastly, this analysis focused solely on the prognostic value of baseline serum calcium, and further investigation is needed to determine whether changes in serum calcium levels during follow-up also predict the incidence of MHO and MUNO.

Obesity and metabolism are intricate processes influenced by numerous factors. Consequently, diagnosing MHO or MUNO requires a broad range of clinical assessments, including measurements of weight, blood glucose, lipid profile, blood pressure, and other metabolic markers. Timely identification of these obesity phenotypes is crucial in preventing, delaying, or mitigating the progression of metabolic abnormalities and associated mortality. Nevertheless, incorporating the diagnosis of MHO and MUNO into routine testing remains challenging. However, our study proposes a compensatory approach by utilizing serum calcium, a commonly accessible indicator, for dynamic monitoring. Significant fluctuations in serum calcium levels can serve as potential indicators for the presence of MHO or MUNO status in both obese and non-obese individuals, prompting further metabolic assessments. This approach holds the potential to enable earlier prevention, diagnosis, and treatment options.

## Conclusions

In conclusion, the results of our study indicate that serum calcium is a valuable indicator of MHO and MUNO risk across different patient groups, with a non-linear association observed. Significant fluctuations in serum calcium levels can indicate the potential presence of either MHO or MUNO, necessitating further metabolic evaluations.

## Supplementary Material

Supplemental Material

## Data Availability

Publicly available datasets were analyzed in this study. These data are available online at https://www.cdc.gov/nchs/nhanes/index.htm.
