# Supplementary material for: Association of serum calcium and metabolically healthy obese in US adults: a cross-sectional study
Source: Ann Med. 2024 Sep 18;56(1):2403721. doi: 10.1080/07853890.2024.2403721 (PMC11411560; doi:10.1080/07853890.2024.2403721)
Supplement: Supplemental Material [file IANN_A_2403721_SM5574.zip › Supplementary Table S1 & S2/Supplementary Table S1 (2).docx]

**Table S1. Associations of serum calcium with MHO and MUNO in NHANES 2003-2006**

| Independent variables | OR | 95%CI | *P* |
| --- | --- | --- | --- |
| Incidence of MHO | | | |
| Calcium | 0.36 | 0.14,0.89 | 0.034 |
| Q1 |  |  |  |
| Q2 | 0.98 | 0.32,3.04 | 0.963 |
| Q3 | 0.55 | 0.19,1.58 | 0.171 |
| Q4 | 0.4 | 0.13,1.24 | 0.081 |
| *P* for trend |  |  | 0.024 |
| Corrected-Calcium | 0.22 | 0.08,0.56 | 0.009 |
| Q1 |  |  |  |
| Q2 | 0.57 | 0.17,1.86 | 0.228 |
| Q3 | 0.33 | 0.11,0.95 | 0.044 |
| Q4 | 0.28 | 0.07,1.12 | 0.061 |
| *P* for trend |  |  | 0.024 |
| Incidence of MUNO | | | |
| Calcium | 2.12 | 1.29,3.47 | 0.011 |
| Q1 |  |  |  |
| Q2 | 0.88 | 0.48,1.63 | 0.556 |
| Q3 | 1.24 | 0.73,2.13 | 0.284 |
| Q4 | 1.7 | 0.88,3.27 | 0.081 |
| *P* for trend |  |  | 0.025 |
| Corrected-Calcium | 2.63 | 1.68,4.12 | 0.003 |
| Q1 |  |  |  |
| Q2 | 1.38 | 0.77,2.47 | 0.175 |
| Q3 | 1.94 | 1.09,3.45 | 0.036 |
| Q4 | 2.32 | 1.18,4.58 | 0.029 |
| *P* for trend |  |  | 0.005 |

Note: Multivariate weighted logistic regression models were adjusted for age, sex, race, education level, family income level, serum ALT, AST, BUN, creatinine, eGFR, Vitamin D3, Dietary calcium intake, smoking status, alcohol intake, physical activity, and CVD.
