# Supplementary material for: Association of serum calcium and metabolically healthy obese in US adults: a cross-sectional study
Source: Ann Med. 2024 Sep 18;56(1):2403721. doi: 10.1080/07853890.2024.2403721 (PMC11411560; doi:10.1080/07853890.2024.2403721)
Supplement: Supplemental Material [file IANN_A_2403721_SM5574.zip › Supplementary Table S1 & S2/Supplementary_Table_S2 (1).docx]

**Table S2. Associations of serum ions with MHO and MUNO in US adults**

| Independent variables | Mode 1 | | | Mode 2 | | | Mode 3 | | |
| --- | --- | --- | --- | --- | --- | --- | --- | --- | --- |
|  | OR | 95%CI | *P* | OR | 95%CI | *P* | OR | 95%CI | *P* |
| Incidence of MHO | | | | | | | | | |
| Phosphorus ^a^ | 0.52 | 0.32,0.86 | 0.01 | 0.38 | 0.23,0.65 | <0.001 | 0.42 | 0.25,0.71 | 0.001 |
| Q1 |  |  |  |  |  |  |  |  |  |
| Q2 | 0.93 | 0.73,1.19 | 0.557 | 0.87 | 0.68,1.11 | 0.259 | 0.86 | 0.67,1.1 | 0.219 |
| Q3 | 1.08 | 0.77,1.5 | 0.662 | 1 | 0.71,1.41 | 0.986 | 1.02 | 0.73,1.44 | 0.888 |
| Q4 | 0.71 | 0.54,0.92 | 0.009 | 0.62 | 0.47,0.81 | 0.001 | 0.64 | 0.49,0.84 | 0.002 |
| P for trend |  |  | 0.02 |  |  | 0.002 |  |  | 0.006 |
| Iron | 1.02 | 1.01,1.04 | 0.001 | 1.04 | 1.03,1.06 | <0.001 | 1.05 | 1.03,1.06 | <0.001 |
| Q1 |  |  |  |  |  |  |  |  |  |
| Q2 | 1.13 | 0.83,1.53 | 0.436 | 1.48 | 1.09,2.03 | 0.013 | 1.52 | 1.12,2.08 | 0.008 |
| Q3 | 1.08 | 0.79,1.48 | 0.623 | 1.58 | 1.14,2.2 | 0.007 | 1.68 | 1.21,2.35 | 0.002 |
| Q4 | 1.39 | 1.06,1.82 | 0.017 | 2.14 | 1.6,2.87 | <0.001 | 2.29 | 1.7,3.08 | <0.001 |
| P for trend |  |  | 0.024 |  |  | <0.001 |  |  | <0.001 |
| Sodium | 0.98 | 0.95,1.02 | 0.335 |  |  |  |  |  |  |
| Potassium | 0.99 | 0.77,1.28 | 0.962 |  |  |  |  |  |  |
| Chloride | 1.13 | 1.09,1.17 | <0.001 | 1.09 | 1.05,1.14 | <0.001 | 1.1 | 1.06,1.14 | <0.001 |
| Q1 |  |  |  |  |  |  |  |  |  |
| Q2 | 1.94 | 1.43,2.64 | <0.001 | 1.71 | 1.26,2.34 | 0.001 | 1.67 | 1.21,2.29 | 0.002 |
| Q3 | 2.22 | 1.59,3.09 | <0.001 | 1.81 | 1.28,2.57 | 0.001 | 1.78 | 1.26,2.53 | 0.001 |
| Q4 | 2.63 | 1.92,3.58 | <0.001 | 2.07 | 1.5,2.85 | <0.001 | 2.11 | 1.54,2.9 | <0.001 |
| P for trend |  |  | <0.001 |  |  | <0.001 |  |  | <0.001 |
| Incidence of MUNO | | | | | | | | | |
| Phosphorus ^a^ | 0.75 | 0.56,1 | 0.048 | 1.31 | 0.96,1.81 | 0.091 |  |  |  |
| Iron | 0.98 | 0.97,0.98 | <0.001 | 0.97 | 0.97,0.98 | <0.001 | 0.98 | 0.97,0.99 | <0.001 |
| Q1 |  |  |  |  |  |  |  |  |  |
| Q2 | 0.9 | 0.76,1.06 | 0.21 | 0.72 | 0.6,0.85 | <0.001 | 0.75 | 0.63,0.9 | 0.002 |
| Q3 | 0.82 | 0.71,0.95 | 0.008 | 0.65 | 0.55,0.76 | <0.001 | 0.67 | 0.56,0.79 | <0.001 |
| Q4 | 0.67 | 0.59,0.78 | <0.001 | 0.58 | 0.49,0.68 | <0.001 | 0.61 | 0.52,0.73 | <0.001 |
| *P* for trend |  |  | <0.001 |  |  | <0.001 |  |  | <0.001 |
| Sodium | 1.01 | 0.99,1.03 | 0.347 |  |  |  |  |  |  |
| Potassium | 1 | 0.87,1.14 | 0.981 |  |  |  |  |  |  |
| Chloride | 0.92 | 0.9,0.93 | <0.001 | 0.95 | 0.93,0.97 | <0.001 | 0.94 | 0.92,0.96 | <0.001 |
| Q1 |  |  |  |  |  |  |  |  |  |
| Q2 | 0.63 | 0.55,0.72 | <0.001 | 0.72 | 0.63,0.83 | <0.001 | 0.73 | 0.63,0.84 | <0.001 |
| Q3 | 0.57 | 0.48,0.68 | <0.001 | 0.72 | 0.6,0.87 | <0.001 | 0.74 | 0.61,0.89 | 0.001 |
| Q4 | 0.61 | 0.52,0.71 | <0.001 | 0.76 | 0.65,0.89 | 0.001 | 0.73 | 0.62,0.85 | <0.001 |
| *P* for trend |  |  | <0.001 |  |  | 0.001 |  |  | <0.001 |

Note: Multivariate weighted logistic regression models with three models to control for confounding factors.

Model 1 was unadjusted;

Model 2 was adjusted for age, race, and gender;

Model 3 was adjusted for age, sex, race, education level, family income level, serum ALT, AST, BUN, creatinine, eGFR, smoking status, alcohol intake, physical activity, and CVD.

^a^ : Model 3 was adjusted for age, sex, race, education level, family income level, serum ALT, AST, BUN, creatinine, eGFR, Vitamin D3, Dietary calcium intake, smoking status, alcohol intake, physical activity, and CVD.
